# Do quality improvement collaboratives’ educational components match the dominant learning style preferences of the participants?

**DOI:** 10.1186/s12913-015-0915-z

**Published:** 2015-06-20

**Authors:** Anne Marie Weggelaar-Jansen, Jeroen van Wijngaarden, Sarah-Sue Slaghuis

**Affiliations:** Department of Health Policy and Management, Erasmus University, Campus Woudestein, P.O. Box 1738, 3000 DR Rotterdam, The Netherlands

**Keywords:** Quality of healthcare, Quality improvement, Program evaluation, Education needs assessment, Competency-Based Education

## Abstract

**Background:**

Quality improvement collaboratives are used to improve healthcare by various organizations. Despite their popularity literature shows mixed results on their effectiveness. A quality improvement collaborative can be seen as a temporary learning organization in which knowledge about improvement themes and methods is exchanged. In this research we studied: Does the learning approach of a quality improvement collaborative match the learning styles preferences of the individual participants and how does that affect the learning process of participants?

**Methods:**

This research used a mixed methods design combining a validated learning style questionnaire with data collected in the tradition of action research methodology to study two Dutch quality improvement collaboratives. The questionnaire is based on the learning style model of Ruijters and Simons, distinguishing five learning style preferences: Acquisition of knowledge, Apperception from others, Discovery of new insights, Exercising in fictitious situations and Participation with others.

**Results:**

The most preferred learning styles of the participants were Discovery and Participation. The learning style Acquisition was moderately preferred and Apperception and Exercising were least preferred. The educational components of the quality improvement collaboratives studied (national conferences, half-day learning sessions, faculty site visits and use of an online tool) were predominantly associated with the learning styles Acquisition and Apperception. We observed a decrease in attendance to the learning activities and non-conformance with the standardized set goals and approaches.

**Conclusions:**

We conclude that the participants’ satisfaction with the offered learning approach changed over time. The lacking match between these learning style preferences and the learning approach in the educational components of the quality improvement collaboratives studied might be the reason why the participants felt they did not gain new insights and therefore ceased their participation in the collaborative. This study provides guidance for future organisers and participants of quality improvement collaboratives about which learning approaches will best suit the participants and enhance improvement work.

**Electronic supplementary material:**

The online version of this article (doi:10.1186/s12913-015-0915-z) contains supplementary material, which is available to authorized users.

## Introduction

Many healthcare organizations are continuously working on a diverse set of improvement projects centred on the triple aim: increasing quality of care, increasing the (evidence based) care outcomes and, at the same time, reducing costs [1]. To achieve triple aim improvements several models and methods from different theoretical backgrounds are used [2, 3]. A Quality Improvement Collaborative (QIC) combines different improvement models and methods. In a QIC, groups of healthcare professionals from different healthcare organizations are brought together to work on the improvement of a specific topic [4, 5].

QICs are described as temporary learning organizations, in which knowledge about quality improvement themes, models and methods for change, is exchanged [4, 6–8]. Integral to the QIC methodology is learning in collaboration with other participants [4, 9]. Most QICs focus on three different learning levels: 1) individual learning from experts in the field of the goal theme and/or the change methodology 2) learning within the network of participating organizations 3) learning within the teams [4, 9–12]. The QIC faculty organizes collective (virtual) meetings to teach team members and support sharing information between different teams [4, 7, 11–14].

QICs are frequently used within Europe, the United States, Canada and Australia and are generally acknowledged for their success [10, 15]. Despite their widespread use, the actual effectiveness of QICs is still in question [5, 16, 17]. Because learning is central to the QIC, more insight into the learning process within a QIC may help to understand how we can improve the effectiveness of QICs. Little research has yet been conducted into if, and how, learning takes place in a QIC [15–17] and how QICs facilitate the learning processes of their participants [18–20]. To understand more about how learning can be enhanced in a QIC it is necessary to gain more insight into how individual participants learn.

Research has shown that people differ in their learning styles [21, 22]. Keefe states that learning styles are “cognitive, affective, and physiological traits that are relatively stable indicators of how learners perceive, interact with, and respond to the learning environment” ([23] p.3). Hence, the match between the learning approach in QICs’ educational components and the preferred learning styles of the participants can influence of the educational effectiveness of QICS.

This article presents the results of a mixed method research of two QICs focussing on improvement of hospital logistics. A survey was used to determine the preferred learning styles of the participants. Next, two logistic QICs were investigated using action research data aiming to explore if and how the learning approach used in the QIC matched the preferred learning styles of the participants and how this affected the learning environment of the QIC. The research question is: Does the learning approach of a QIC match the learning style preferences of its individual participants and how does this affect the learning process of the participants?

## Background

### Learning styles

To gain more understanding of the learning styles of the QIC participants, theoretical knowledge on different learning style models was used [21–34]. A systematic review [21–24] identified 71 learning styles models from different theoretical backgrounds: psychology, sociology, business studies, education, management and policy. The reviewers divided the learning style models into five ‘families’, each of which emphasizes a different paradigm of learning styles [25, 26]:

1. learning styles models which reflect a perception that learning styles are largely constitutionally-based including the visual, auditory, kinaesthetic and tactile modalities;
2. learning styles models which reflect deep seated features of the cognitive structure of individuals including patterns of ability and needs;
3. learning styles models which reflect a perception that learning styles are one component of a relatively stable personality type and therefore use methods to assess individuals’ personalities in combination with learning, such as Myers Briggs Type Indicator [28] and Jackson’s Learning Styles Profiler [29];
4. learning styles models which aim to measure flexible or stable learning preferences of individuals (over time), such as the Learning Style Inventory [30], Learning Styles Questionnaire [31] and 4MAT [32];
5. learning styles models which are linked to learning approaches, strategies and orientations, which pay a greater attention to personal factors, such as motivation, the influence of environmental factors and cooperative learning.

Because our research focused on individual learning styles related to collective learning processes and a large amount of learning activities a model from Family 5 seems appropriate to study preferences in the context of quality improvement work [33, 34]. We used a relatively new, but validated, learning style tool from Family 5, based on the model of Simons and Ruijters [35]. The model was developed in a study that combines learning styles and paradigms about organizational change [36] and therefore fits well in the aims of our QIC study. In this model five different learning styles are distinguished [35] (see Table 1).

**Table 1:** Learning Styles of Ruijters and Simons [35, 36]

Acquisition	Gathering objective knowledge (facts, theories) from experts; learning is guided by achieving a concrete result. Examples of relevant learning environments are classroom lectures, documentaries and literature study.
Apperception	Observing others/examples and to imitate what works; learning from observing experienced role models and best practices. Learning under pressure, such as hectic, relatively unpredictable and constantly changing work environments. Examples of relevant learning environments are real world situations, such as site visits, shadowing and demonstration.
Discovery	Jumping into new and interesting issues based on personal curiosity and fortuitous circumstances and reflecting on the experience with sagacity to discover new insights; learning and life are combined and must be interesting and inspirational. Learning is based on self-reflection and focused on knowledge creation. Examples of relevant learning environments are practical assignments, brainstorming, storytelling and open space conferences.
Exercising	Practising through supervised repeated exercises in a safe ‘laboratory environment’; learning takes place in training sessions which recreate realistic situations and provide the opportunity to practise new skills. Examples of relevant learning environments are role-play, simulations, workshops and skills labs with an experienced teacher to point things out or pass on knowledge.
Participation	Engaging in a dialogue or discussion with others to share opinions and sharpen ideas; learning is a social event involving interaction and communication (learning from and with others). These dialogues and interactions require equality and trust among participants. Examples of relevant learning environments are peer consultation, communities of practice and case discussions

### Learning styles in QICs

In this paragraph we first review the literature on the combination of quality improvement work and learning styles. Next, we elaborate on the connection between QIC’s and learning styles.

Some research has already been carried out on how quality improvement work in healthcare is linked to learning [37–39]. Murry and Chapman [37] highlight in their study four dimensions necessary to activate the quality improvement cycle: 1) developing capabilities, 2) generative learning, 3) adaptive learning matched to the situation and 4) learning styles. In addition, the effects of inter-professional education on improvement were studied in a Cochrane systematic review [39]. However, due to lack of evidence (only six studies could be included) no solid conclusion could be drawn.

Nadeem et al. [17] performed a systematic review on the different QIC components and how they relate to improvements in professional or patient level outcomes. Nadeem et al. [17] have identified 14 crosscutting QIC components which encompass specific educational components, such as learning sessions, phone meetings, training in QI methods and teaching strategies to foster cross-site collaboration. Nadeem et al. [17] conclude that only in a very few studies a description of the educational components was available, allowing insight into the QICs’ learning processes. Hence, there is little evidence on the critical features of the educational components of QICs. We found only four studies [40–44] that mention the benefits of different QIC components with regard to the participants’ learning processes. Freemont et al. [40] conclude that learning sessions with experts and peer support are seen as helpful. Leape et al. [41] show that the results on improvement goals increased when more team members attended the learning sessions. Nembard [8, 42] adds that the results also increased when more QIC components were used in the teams, in particular learning sessions and monthly reports. Gustafson et al. [43] conclude that learning sessions and interest circle conference calls delivered fewer improvement results compared to coaching. Thus, there is inconclusive evidence for the contribution of different QIC components to the learning processes of participants and the improvement results. Nadeem et al. [17] conclude that despite the fact that many studies acknowledge the importance of learning processes in QICs, it appears that research on the combination of learning styles and the learning approach of QICs is currently lacking and there is a need for more insight.

## Methods

In this mixed methods research we combined a questionnaire study of learning preferences with an analysis of action research data.

### Setting

We studied two QICs focussing on improving logistics in hospitals. One QIC aimed to reduce access time to outpatient clinics by using the principles of Advanced Access [44]. The other QIC was focussed on reducing throughput time for patients by at least 20 %, by developing clinical pathways and/or using the principles of Process Redesign [45]. Both QICs were part of Faster Better, a QIC program across the Netherlands striving to improve the quality of Dutch hospitals. Both the logistics QICs used the Breakthrough approach [11, 12] and were organized in the same way. Ethical permission for this study was not necessary under Dutch law as no patient data was collected. Every participant in both QICs was informed about the study and gave approval for using the data.

### Survey study

#### Measurement instrument

To assess learning styles a questionnaire developed by Ruijters and Simons [35, 36] was used. See for the Learning Style questionnaire of Ruijters [36] Additional file 1. This learning style questionnaire measures the preference for the five different learning styles environments. The questionnaire consists of 15 questions. For example: “How do you deal with errors?” or “Which competence should the ideal supervisor have?”. For each question, four or five statements based on the five learning styles were presented. For example:

“What circumstances helps you to develop?

- Complex issues which must be resolved at short notice (learning style apperception).
- An inspired meeting with others (learning style participation).
- Environments in which many knowledge sources are present (learning style Acquisition).
- When there is time and space for practising (learning style Exercising).
- In work situations where I can come across new interesting issues (learning style Discovery)” [36].

Some statements are relevant for two learning styles and therefore contribute to both learning style preferences. The respondents scored each statement (in total 65 statements) on a five-point Likert scale: ranging from “not applicable to me” (1), “average” (3) to “fully applicable to me” (5).

For each learning style, measurement properties were assessed (see Table 2). Correlations analysis with the five learning styles revealed that the scales in general are positively and high to moderately related, *r* ranged between 0.23 and 0.76. The results were similar to the internal consistency analysis reported by Ruijters [36].

**Table 2:** Descriptive statistics and correlations for the five learning style sumscores

	Apperception	Participation	Acquisition	Exercise	Cronbach alpha
Apperception					.57
Participation	.51**				.68
Acquisition	.31**	.54**			.72
Exercise	.38**	.65**	.76**		.67
Discovery	.51**	.43**	.23*	.33**	.64

Legend:

**significant for p < 0.001

*significant for p < 0.05

#### Participants

The questionnaire was distributed among all project members of 28 project teams of eight hospitals during the last plenary meeting of each QIC. Project team leaders were asked to distribute the questionnaire among those not present. A total of 170 questionnaires were distributed; 92 among the Advanced Access participants and 78 among the Process Redesign participants. The questionnaire could be returned anonymously. 142 Questionnaires were returned (83.5 %). In our analysis a project team was included if at least 75 % of all its team members responded; 23 project teams (82.1 %) were included in our study. The final sample for analysis included 12 of the 15 teams (80 %) for Advance Access and 11 of the 13 teams (84.6 %) for Process Redesign. The final sample was N = 125, resulting in the following response rates of 125/142 = 73.5 % in total, with N = 72 for Advanced Access (75.8 % response) and N = 53 for Process Redesign (70.7 % response).

#### Analysis

Because the educational components and learning approach of both QICs were organized in the same way [10, 11] the data sets could be combined. Nevertheless, an independent samples *t*-test between the two logistics QIC was performed. This revealed no statistically significant differences between participants and their preferred learning style in the two different QICs. Therefore, there was no restriction to studying the group of respondents from both QICs as a whole. Analysis was performed with SPSS 19.0 software and consisted of three steps. First, the sample characteristics of the two QICs were analysed using descriptive statistics. Next, based on the learning style inventory scores, two variables were constructed. First of all, for each respondent, learning style sum scores were computed based on the learning style sub-scale results for the 15 questions. In addition, the learning style sum scores were ranked to identify learning style preferences for each respondent (rank 1–5). Based on these ranked scores, frequencies and percentages for the total sample were computed to indicate the extent to which the styles were preferred. Last, we explored the potential effects of differences in gender, age, professional background and project role in connection with the ranked learning style scores with ONEWAY ANOVAs; post hoc tests were computed with the Tukey’s honest significant difference (HSD) procedure for pairwise comparisons of the means.

### Action research

This part of the study aimed to gain a deeper insight into the match between the learning style preference and the learning process of the participant. Research was focused on four different aspects: 1) how the learning approach of the educational components of the QIC match the preferred learning styles; 2) how the educational components are perceived by the participants; 3) how this influences their learning process and how the QIC contributed to increased knowledge and skills for working on quality improvement; 4) how this influenced improvement work within the participants’ hospitals. These four aspects must be studied as a coherent and influencing system within a context [46–48]. Thus the four aspects were not studied as independent questions; rather we took an integrated approach seeking to discover relationships. To obtain in-depth information, a study based on action research traditions was performed. Action research is a process-oriented research methodology where the researcher participates in the routine practices of their ‘study objects’ [49, 50]. Two authors were assigned as advisors to four hospitals participating in the studied QIC. By participating in the QICs’ educational components and real-life situations in the hospitals the researchers had the opportunity to observe what participants of the QICs actually do, instead of what respondents in an interview or questionnaire say they do. Argyris [50–52] describes this as the difference between espoused theory (what people say) and theory in use (what people actually do).

By nature action research is the ideal methodology for identifying and improving practices in healthcare [53, 54]. Action research is commonly designed into five cyclical phases starting with Diagnosis and ending with Learning and Refinement [55]. In this study we only performed the first two phases: Diagnosis (identify and define the problem using a variety of data collection methods) and Action Planning (consider courses of action). The phases Implementation, Evaluation and Learning were not possible, because we were not the QIC program leaders and performed the analysis after the QIC was finished.

Data collection: In the advisor role two researchers were present at the studied educational components and at more than 100 meetings within the four hospitals. Close observations [56–59] during these meetings were documented in a daily reflective project journal [59–61] with chronological descriptions and observations of facts and systematic reflections. The facts concerned the QICs’ educational components, project and hospital meetings. In this project journal systematic reflections were also made on topics which were surprising or intriguing [60, 61]. In addition, the researchers wrote minutes of conversations. These minutes were summarized in thick descriptions [57, 58] about the opinions of hospital employees and faculty about QIC learning approaches and educational components.

#### Analysis

The project journal, reflective notes, minutes, and thick descriptions were analysed by open coding. These initial analyses identified four themes: 1) the QIC components as a temporary learning organization, 2) how the transfer of knowledge and skills progressed, 3) via which learning style this transfer occurred and 4) which aspects or conditions of the QIC educational program were experienced as a help or hindrance in the learning processes.

Next, the initial findings were shared with one professor in Operations Management and two QIC program leaders in two sense-making meetings [62]. The intention of the sense-making meetings was to share thoughts and beliefs about the QICs openly and on equal terms [62, 63]. Themes derived from the analysis were discussed. In this way researchers were able to expose a general analysis, test their assumptions and interpretations of the data [63, 64], and reflect on issues to generate actionable knowledge about the match between learning styles and the learning approach used in the QIC educational components.

## Results

First, the results of the learning style survey are presented. Second, the results of the action research are described.

### Questionnaire survey sample characteristics

The sample comprised a variety of the major hospital professional groups: medics (e.g. physicians, fellows and residents), nurses (e.g. registered nurses, student nurses and nurse practitioners), allied health care staff (e.g. ambulatory physician staff, respiratory, physical and occupational therapists, dieticians and pharmacists), administrative employees to support care planning, management and other support staff (e.g. advisors and policy makers). Half of the respondents were management and other supportive staff (almost 50 %), the other half were frontline professionals. The variables gender and age of the professionals comprise an average selection of hospital staff when compared against figures from 2008 from the Dutch Association of Hospitals website [65] and show similarities with these figures. See Table 3 for an overview.

**Table 3:** Characteristics of Respondents (N = 125)

Gender	Male	32 %
	Female	68 %
Age	<30 years	12.8 %
	31 to 40 years	26.4 %
	41 to 50 years	44.0 %
	>51 years	16.8 %
Professional background	Medics	19.2 %
	Nurses	11.2 %
	Allied Healthcare Staff	3.2 %
	Administrative employees	12.0 %
	Management	28.8 %
	Support staff	20.8 %
	Other	4.8 %
Project team’ role	Project leader	20.0 %
	Project team member	54.4 %
	Support staff	17.6 %
	Other	8.0 %

### Dominant learning styles based on the questionnaire

As indicated previously, two variables were constructed for learning styles: individual sumscores per style and ranked scores (see Table 4). The analysis of the preference for the calculated sum score and ranked learning style revealed that the most preferred learning styles of all QIC participants (N = 125) were Discovery (calculated: M = 13.5, SD = 5.49; ranked: for 48.4 % of participants, thus this style had the highest sumscore) and Participation (calculated: M = 13.3, SD = 5.01; ranked: 34.9 %). Both learning styles focus on learning within a social context with other people and combine ‘real life’ experiences to learn. Discovery focuses more on individual insights whereas Participation focuses more on collectively gained insights.

**Table 4:** Frequency counts and ranked percentage

ranking	Apperception		Participation		Acquisition		Exercising		Discovery	
	freq.	%	freq.	%	freq.	%	freq.	%	freq.	%
1	10	7,9	44	34,9	26	20,6	3	2,4	61	48,4
2	19	15,1	47	37,3	28	22,2	6	4,8	31	24,6
3	35	27,8	24	19	23	18,3	23	18,3	20	15,9
4	21	16,7	10	7,9	32	25,4	49	38,9	9	7,1
5	41	32,5	1	0,8	17	13,5	45	35,7	5	4

Legend 1 = most preferred learning style, 5 = least preferred learning style

The least preferred learning styles of the QIC participants were Exercising (calculated: M = 7.5, SD = 5.93; ranked: 35.7 %) and Apperception (calculated: M = 8.8, SD = 4.77; ranked: 32.5 %). Both of these learning styles necessitate dedicated time for learning activities. While Exercising requires a safe learning environment, the Apperception learning style benefits most from some excitement and tension in daily practice.

An exploratory analysis of the differences in learning style preferences was performed. Various ONEWAY ANOVAs tested whether differences in age, gender and professional background and project role were associated with a different sum score for each learning style.

#### Gender

**Fig. 1:**
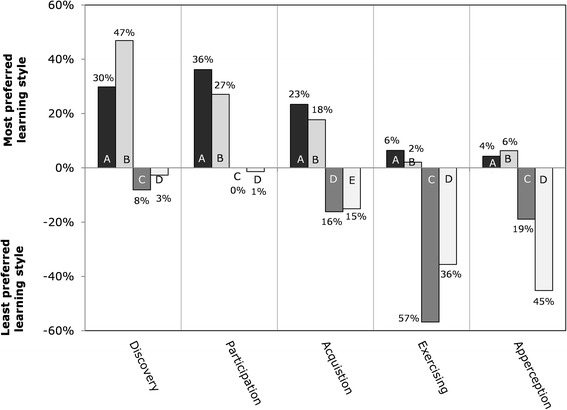
Percentage of the most and least preferred learning styles by gender (N = 125). Legend: A = most preferred by Male; B = most preferred by Female; C = least preferred by Male; D = least preferred by Female

The only marginal significant difference found in gender was in the Discovery learning style (F(1,124) = 3.64, *p* = 0.059). Males (M = 12.2, SD = 5.5) were less inclined to prefer this learning style compared with the females (M = 14.1, SD = 5.4). Men seem to have a slightly greater preference for Participation (see Fig. 1). The least preferred learning style for women was Apperception and for men Exercising.

#### Age

The differences between four age groups (<30; 31–41; 41–50; >50 years old) revealed only significant differences for the learning style Acquisition (F(3,120) = 3.113, *p* = 0.03). The main difference concerns the age group 31–40 years (M = 8.4, SD = 6.1) and > 50 years (M = 13.4, SD = 7.5); the latter age group has greater preference for the learning style Acquisition.

#### Project role

The analysis shows mostly similar preferences between project leaders and project members, except for a slight difference in preference for Exercising (F(2, 113) = 3.464, Tukey HSD *p* = 0.035).

#### Profession

**Fig. 2:**
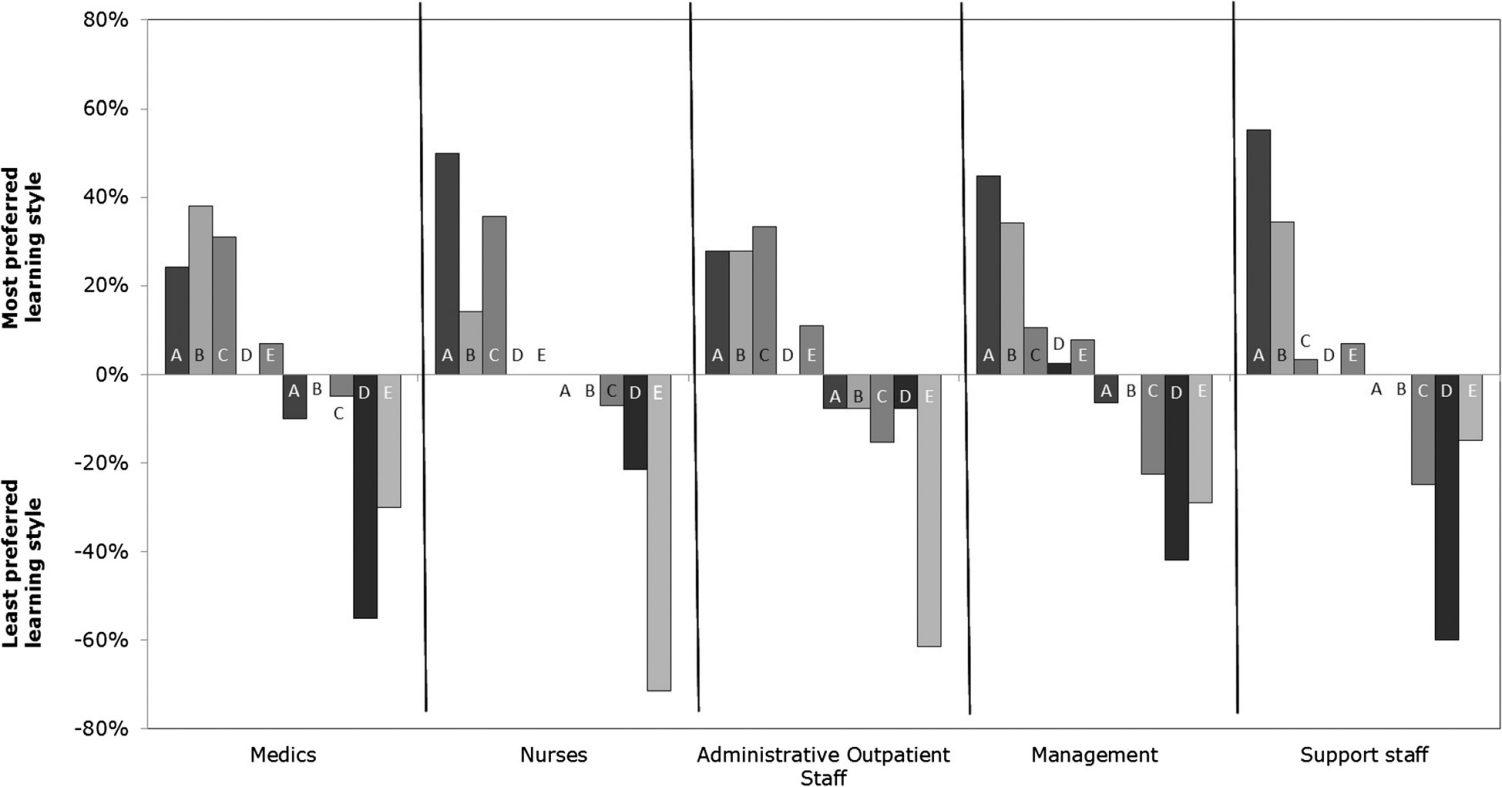
Percentage of the most and least preferred learning styles by profession (N = 125). Legend: A = Discovery; B = Participation; C = Acquisition; D = Excercising; E = Apperception

Looking at the results presented in Fig. 2 which illustrates the percentages of the ranked preferences in the different professional groups. There were several notable distinctions between the different professionals in their ranked preferences. Medics preferred Participation (M = 13.3, SD = 6.1) scored this as the most preferred learning style. Advisors (M = 14.8, SD = 5.8), nurses (M = 16.8, SD = 5.1) and management (M = 14.1, SD = 5.6) preferred Discovery. Administrative outpatient staff preferred the most Acquisition (M = 12.6, SD = 5.1). The learning style Acquisition and Discovery overlap with the focus on the body of knowledge of what needs to be taught.

The least preferred learning style for medics (M = 7.0, SD = 6.5) and advisors (M = 5.2, SD = 5.5) was Exercising and for nurses (M = 8.9, SD = 5.0) and administrative outpatient staff (M = 8.7, SD = 4.6) Apperception. This is a noteworthy result, because Apperception and Exercising are learning styles which build on the experience of the ‘teacher’.

### QICs and learning styles based on the action research data

In this paragraph we present the findings of the action research data on how the match between the learning approach of the QIC educational components and participants’ learning style preference influenced the learning processes. We describe the educational component of the QICs and which learning style approaches were used (presented between brackets). We also describe how the participants experienced the QIC educational components.

The two logistics QICs studied have a similar set up to most QICs using the Breakthrough Approach [5, 11, 12]. The project teams of different hospitals work on specific topics and well defined goals, derived from scientific and research based knowledge (e.g. clinical guidelines). A faculty of clinical professionals who are experts on the subject *and* experts in quality improvement methods supports the project teams. The extensive use of multiple small test improvement cycles accompanied by measuring achievements and reflection on actions is the main improvement approach [10, 66, 67]. The most important QIC educational components for both QICs were:

- Four national conferences for all project team members of the QICs, where guidance and instruction was provided by experts. Half-day learning sessions, for project leaders and advisors, organised on a quarterly basis, where results were presented (sometimes by benchmarking) and successes and barriers were discussed.
- One hospital site visit by the QIC faculty, to exchange ideas and reflect on the lessons learned within each hospital.
- List-serv, an online tool only assessable for the participants, to store written information and send secured e-mail [68].

In addition, a leadership network conference for all CEOs and leading consultant clinicians from each hospital was organized. The aim of this conference was to transfer information about logistics improvement and change management approaches and to explain the importance of their supporting role.

#### Four national conferences for each QIC

The national conferences consisted of different elements: plenary lectures, time for the teams to work on their projects and cross-team learning activities such as exchanging experiences and ideas [11, 12].

During the *first two* national conferences almost all team members attended. Both conferences for both QICs had the same set up: five lectures were given to explain what the teams should do (Acquisition). Next to that, a physician who had already successfully conducted the improvement project relayed his experience in the form of a narrative (Apperception). After the first national conference the team members stated that they had a clear view as how to start their project.

“We need to start by defining a goal and start assessing the current situation in the next week. We need to discuss the indicators and involve B. [employee of the financial department, responsible for extraction of data].” (administrative outpatient staff hospital B, Advanced Access QIC)

The project team members felt they had acquired enough knowledge (Acquisition). However, after the second national conference different team members expressed some disappointment. They again expected a clear set of instructions (Acquisition), but felt they did not receive that. At that point the multiple small test improvement cycles (Plan-Do-Study-Act) to experiment were starting, and they expressed not feeling sufficiently confident to proceed.

“Without clear ‘homework’ about how to proceed we feel lost. Please can you help us and give some directions, otherwise we will lose so much time trying to find our way.” (manager hospital C, Process Redesign QIC)

The team members explained that they did not have problems with understanding the principles of performing small test improvement cycle experiments, but with the change management aspect of this job. The experiments require their having to involve their co-workers, teaching and motivating them. Therefore they need to organise meetings to share insights and solve problems collectively (Participation). However they had little or no experience with this and needed guidance on how to interact with their colleagues

During the *third and fourth* conferences the number of team members attending declined. Particularly medics and management skipped these meetings. The reason for this, they expressed, was that they could not learn anything new at the meetings. The benefits gained were too few compared to the time and effort spent:

“The shared information is identical and the QIC faculty cannot offer any solutions to the current issues I face in my outpatient clinic. We have to do this on our own and I do not expect them to have the magic key with all the answers…. The nurse will go and she can share relevant new information in the project meeting.” (physician hospital C, Advanced Access QIC)

This quote highlights the fact that team members felt that the educational components of the second conference and the program of upcoming conference (mainly Apperception and little Acquisition) no longer matched their preferred learning style (Discovery and Participation).

The third and fourth conference started with an open space session in which everyone could view posters displaying the results achieved by each team (Apperception). After this, lectures by the faculty on the next step in the project were held (Acquisition) and a narrative talk by an experienced expert medic was given (Apperception). We observed that some teams continued working on their projects instead of attending these lectures in the conference room.

“We already know what they will talk about; there is nothing new to learn.” (physician hospital A, Advanced Access QIC) “We have too much work and we need to make progress, so we prefer to use this time on our project.” (nurse hospital D, Process Redesign QIC)

The first quote illustrates how the Acquisition learning style fails to match participants’ preferences. Team members were looking for new knowledge but didn’t find the lectures or talk interesting enough. Some team members pointed out that the subject of the lectures didn’t relate to the problems they were facing at that moment.

#### Quarterly half -day learning sessions and faculty site visits

Four half-day learning sessions were organised specifically for project leaders (mostly physicians) and support staff (e.g. advisors). In these sessions a substantial amount of time was spent on the results of the projects based on the indicator measurements (learning style Acquisition). Research on QICs shows that motivation is aligned with being able to observe concrete positive results arising from the improvement work [4, 14]. With this in mind, the achievements of each project compared to the national set goals were shown. However, the project leaders stated that, despite the importance of the national goals (because QICs are funded by the government), these goals are not always considered important by the project team members. Rather, teams adapted these goals to fit their local context, whereby team members took into account what was feasible and desirable (Participation). This resulted in difficulties in the standardized gathering of objective and comparable information about all the QIC projects (Acquisition), and benchmarking therefore became challenging (Apperception).

During site visits the formal national set indicators were the focal point and a central theme in the communication between the QIC faculty and the board of the hospitals. If a team was not making substantial improvements based on the main indicator within the prescribed timeframe, some faculty members deemed the project a failure. In contrast, most project team members felt they had done an excellent job and had made great improvements, even if the data did not suggest this. Consequently, CEOs expressed their disappointment about the gap between presented data and local experience. They felt that the data could be more suitably used in a dialogue about figures and ratings (Participation) rather than as a form of evaluation. A dialogue between project team leaders, hospital management and QIC faculty (Participation) would provide the opportunity to share reasons why the teams had not reached the national set goals.

“Let’s give you an example: The team managed to decrease the throughput time for diagnosis for patients with suspected colon cancer from one week to one day. By achieving this goal, we [the hospital] received an increasing number of referrals from nearby hospitals. Consequently our throughput time actually rose… It is a shame that this project is now seen as a failure.” (CEO hospital B)

We noticed that this particular team was in the process of creating new knowledge about how to deal with an increasing flow of patients (Discovery). They were willing to share this knowledge (Apperception), but because of the faculties’ rigid framework they felt not encouraged to do so.

#### List-serv

The List-serv is an online tool for the storage of documents and supports interaction between the QIC participants [68]. The QIC faculty used the List-serv to disseminate program documents and progress reports. The List-serv was introduced as a communication channel to encourage participants to exchange ideas (Apperception and Participation) and to provide tools (Acquisition). Also, the List-serv had a chat function to steer sessions in discussing problems and solutions (Participation). Surprisingly, in practice the List-serve was only used by the teams as an archive for documents.

“I don’t know the people at the other end of the line, and therefore I don’t want to ask for help” (outpatient nurse hospital C, advanced access QIC).

#### QICs in general

The QICs program leaders described the aim of the QICs from a learning perspective in three ways. The first aim concerns the transfer of knowledge about the goal and change package of the QIC (Acquisition). The second aim is to encourage the mutual exchange of experiences and with that, the diffusion and dissemination of information throughout the QIC teams (Acquisition). The third aim involves the formation of a learning network, in which participants both contribute and receive information (Participation). The QIC faculty expressed that these three aims were not so easy to achieve, because the QIC participants did not form a homogeneous group; the project team members differed in profession, work experience and experience of improvement projects. This led to difficulties in finding a good balance in the level of knowledge offered. Some of the project team members felt they could not gain enough new knowledge (Acquisition), especially during the national conferences and by using the List-serv. As a result a substantial group of participants no longer attended the national conferences and did not use the List-serv as a tool to share information. Unfortunately, their absence further decreased the potential to make considerable contributions to the knowledge transition (Acquisition) and new knowledge development (Discovery).

An important element of the improvement methodology in QICs used to change daily practice is the multiple small test improvement cycle experiments methodology. These experiments fit very well with the most preferred learning style, Discovery: just jump in, have a go and try something new! However, we noticed that teams were slightly reluctant in starting to experiment, but were more engaged in an implementation approach. The written change package and the lectures were very clear with concrete steps or activities that will contribute to the improvement (Acquisition). For instance, the ten principles of Advanced Access or the five steps to reduce the throughput time.

“We hesitate to start small rapid cycle experiments, and by this learning how to improve. I cannot convince them to work on both the short term based on the required goals, and the longer term improvement of their competence for improvement work” (advisor hospital D, process redesign QIC)

## Discussion

The first part of our study focused on the question: Does the learning approach of the QIC match the dominant learning style preferences of the participants? The learning style survey showed that the most preferred learning styles were Discovery and Participation. Only slight differences between participants based on age, gender, professional background and project role were found. Specifically, the preferred learning style of administrative outpatient staff and participants younger than 50 years old was identified as Acquisition. Discovery and Participation learning style require learning environments in which giving meaning and sense-making by reflecting on one’s own experiences are important. These findings correspond with other studies on learning in relationship with improvement work. Scott [69] found similar results in his systematic review about the effectiveness of improvement strategies. One of the most effective quality improvement strategies is professional education in interactive small groups focussed on cases (over 10 % absolute increase). Moreover, Minkman et al. ([9] p.10) conclude in their research on a stroke QIC that Participation was important: “the possibility for exchanging ideas and results with other regions were motivating factors, which emphasized achieving results.” Our survey showed that the learning styles Apperception and Exercising, both focussing on experience-based learning, were least preferred.

The second part of our study focused on the question: How does the match between the preferred learning styles and the QICs’ learning approach affect the learning process of participants? We conclude that the way in which the QICs were organised did not sufficiently suit the preferred learning styles Discovery and Participation; in fact, the lesser preferred learning styles Acquisition and Apperception formed the QICs’ central learning approaches. Our research also showed that what the QIC offered was perceived differently over the course of time. In the first meetings faculty lectures (Acquisition) and expert peers’ narratives (Apperception) as learning approach were highly valued as an efficient way to gain sundry knowledge about the upcoming improvement work. However, later on the participants expressed a greater need for interaction with others and the opportunity for reflection on their situation, which are elements of the Participation and Discovery learning style [35, 36]. The greatest concern among participants was whether the lecture themes could really be applied in their practice; they felt the content was not focussed on ‘the real thing’.

In our theoretical framework our starting point was the idea that people have different (preferred) learning styles [25–27]. Our findings confirm a common implicit notion of learning styles: learning will be less effective or at least modestly efficient if educational components do not fit the (preferred) learning style of the participant [26]. However, little evidence is available to support this argument [26]. To our knowledge, this research is one of the first to explore this assumption empirically in the context of quality improvement in health care.

Authors reflecting learning style family one, two and three believe that learning processes and learning style preferences are relatively stable (constitutionally based: cognitive structure and ability, personality type) [25–27]. Applying this perspective to our findings one could argue that a QIC might be more effective if the learning approach fits the preferred learning styles Discovery and Participation. Moreover, we wonder to what extent the absence of this match poses difficulties for the transfer of knowledge and skills. Perhaps realistic situations that reflect every day practice, such as site visits and training on-the-job, would be more suitable learning approach for QICs, because they contribute to the learning style Discovery. In addition, peer to peer consultation about the most challenging and sensitive issues in improvement, and dialogues between experienced project leaders and/or leading consultant clinicians of successful projects and project team members, could be offered to strengthen Participation. We found some support for these ideas in the quantitative research of Gustafson et al. [43] on educational components of QICs that showed that interest circle calls yield significantly better results than learning sessions. Furthermore, more peer to peer learning and networking was also seen as helpful in the research of Fremont et al. [40]. However, because this was not offered in the QIC studied we cannot be certain about this.

In contrast, authors reasoning from learning style families four and five [25–27] suggest that learning style preferences are also driven by context and content and can change over time [22, 23]. The satisfaction of the participants with the Acquisition and Apperception educational approach at the beginning of the QIC and the dissatisfaction with these learning styles later on may also be explained by different influences of the content and context that the program entailed. While some learning style-related behaviours may depend on the specific context in a team, still it is striking that many participants ceased participation in the QIC, especially if the educational components did not provide enough new insights in the eye of the participants. Gaining new insights is closely connected to the way people learn specific content and the context in which this is offered, and therefore the learning approaches of the QICs influence this.

Numerous reports about quality improvement curricula exist in the literature [70–72]. Yet, only a small number of articles describe the actual educational methodology of these curricula and what participants learned (knowledge and skills). Similarly, research on how this affects their improvement work is lacking [17, 19, 20]. At this stage unfortunately, we cannot validate our findings with empirical research of others. We would strongly welcome further work on the interplay between the participants’ learning styles, learning approaches in QICs’ educational components and how a QIC can be geared to facilitate the improvement processes.

Next we consider some limitations of the methods used in our research. First, the learning style questionnaire utilized is not commonly used. Although it has been validated in previous research [35, 36] there is not much evidence on the applicability of the model for this type of research. Also, the tendency to give socially desirable and acceptable answers in a self-assessment survey and the creation of answer tendencies is always a possibility. Nevertheless, we did obtain a strong data sample using a theory based validated questionnaire. Finally, we recognize that in our theoretical framework the main focus is on individual learning style preferences and the learning approach of educational components in QICs; less emphasis is given to collective learning processes and how the team level performance and learning may interact with content and/or context. Future research could extend the knowledge in this direction.

Second, action research performed by researchers who also have the role of hospital advisor could be considered problematic in terms of validity. Politics, dynamics, ethics and context issues which influence its emergent process are embedded in data collection based on action research methodology traditions [73]. Being aware of this, we used triangulation between observations in our research diary, written minutes about conversations and thick descriptions. The sense-making meetings were also an attempt to confirm the findings in a rigorous way [53, 56]. Action research is by nature a cyclical process in which practice is influenced by research findings. This was not the case in our study, because we analysed our action research data and performed the learning style survey after the QIC was finished. We therefore invite other QICs to use the results of our clinical inquiry [60].

In both QICs and in action research the emphasis is on the development of organization through learning [4, 52, 58]. Both recognize the importance of building knowledge on what works within this specific context by engaging the ‘study subjects’ in research and empowering them [74]. Until now no research has been carried out on the relationships between expanding knowledge and skills and the results of a QIC in terms of improvement aims. We call for more research on learning approaches and educational components of QICs in order to gain more knowledge on how QICs contribute to improvement work in the longer term.

## Conclusion

QICs are used by various organizations seeking to improve healthcare. Despite the popularity of QICs, they are described as a ‘black box’ in terms of their effectiveness [75] and especially their contribution to the development of skills among healthcare professionals [17, 76]. In this research we studied the preferred learning styles of participants using a learning style survey and concluded that the most preferred learning styles were Discovery and Participation. Only slight differences between participants based on age, gender, professional background and project role were found. The learning style Acquisition was modestly preferred and Apperception and Exercising the least preferred. However, the educational components of the QICs studied (national conferences, half-day learning sessions, faculty site visits and use of List-serv) mainly employed the learning approaches Acquisition and Apperception. With action research data we could elucidate the participants’ perceptions of the learning approaches of the QIC educational components. Our evidence suggests that the participants’ satisfaction with the educational approaches offered changed over time. Lectures provided by the QIC faculty (Acquisition of knowledge) and narratives from experienced peers (Apperception) generated enthusiasm and motivation to change in the beginning. Later on the QIC participants were less satisfied with the educational components offered; perhaps the more preferred learning styles Participation and Discovery would be more suitable and conducive to true learning.

The outcome of this study provides guidance for future organisers of QICs with regard to which learning approaches will most benefit the participants. In addition, if participants know their preferred learning style, they could be more aware of and responsible for their own learning path. QICs serve to improve the quality of care by facilitating learning of the participants. The question is how QICs can strengthen learning processes of individuals and foster learning in teams and on organizational level. More research is needed in this direction.

Quality improvement evaluations are often confined to outcomes and results. We would welcome a study of learning environments organized on the basis of the preferred learning styles Participation and Discovery and how this influences the outcome of improvement projects. In contrast, evaluation research could be expanded to include the learning processes of the participants, studying the learning content (what is taught), learning style (preferences) and the form of the educational components (how it is taught). Moreover, the type of quality problem may mediate the relationship between learning styles and the educational components needed. Future research could address how the learning styles might be associated with different quality problems and improvement processes.

## Additional file

Additional file 1:
**Learning Style Questionnaire of Ruijters.**
