# Supplementary material for: Do quality improvement collaboratives’ educational components match the dominant learning style preferences of the participants?
Source: BMC Health Serv Res. 2015 Jun 20;15:239. doi: 10.1186/s12913-015-0915-z (PMC4473844; doi:10.1186/s12913-015-0915-z)

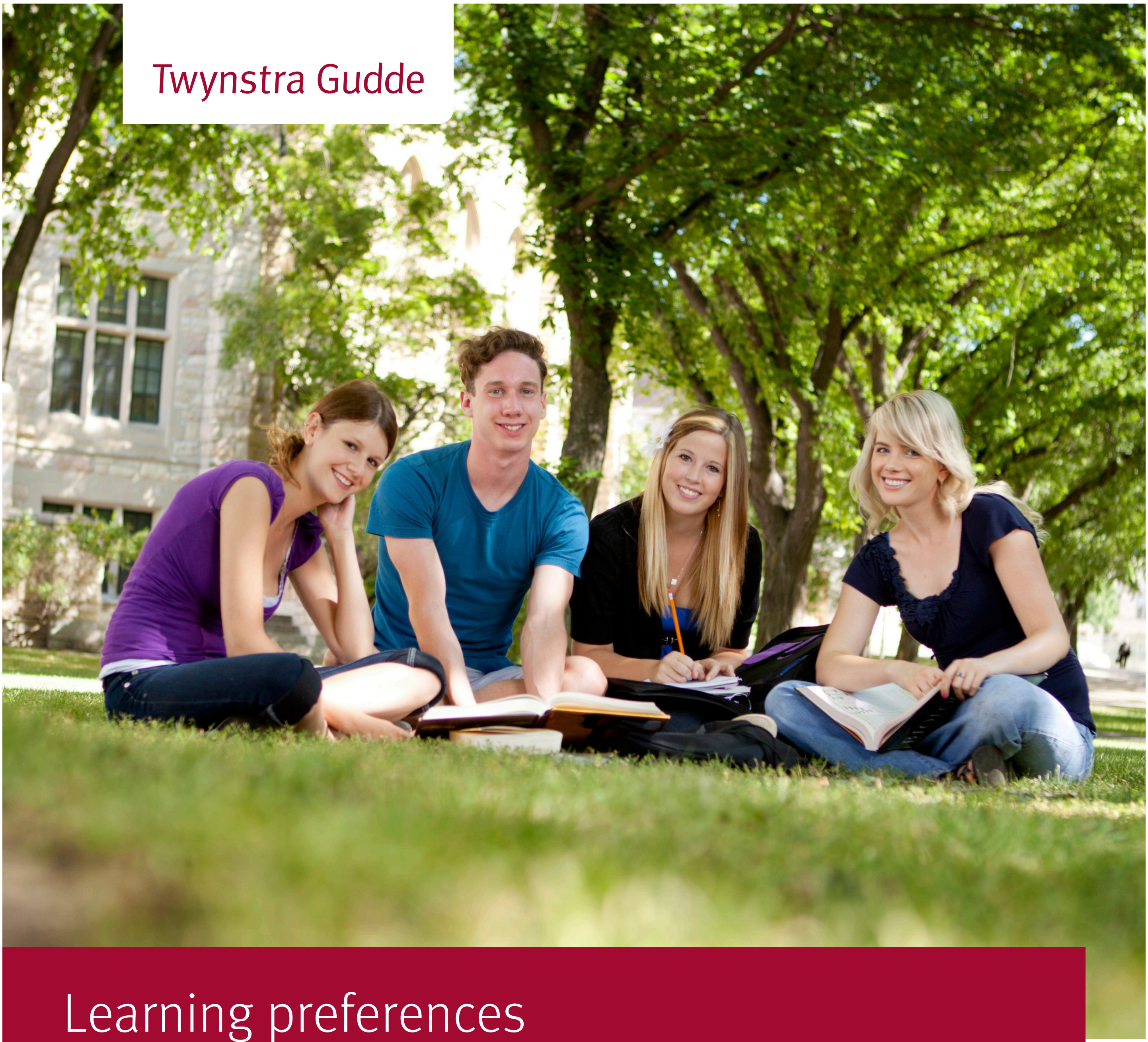

Twynstra Gudde

Learning preferences

Language of Learning

Date ______________________

Name ______________________ M/F Age ______________________

Job title ______________________

Organization ______________________ Department ______________________

Number of years experience ______________________

Education ______________________

Main subject ______________________

Information from this scan will be used to research the learning preferences. This information will be anonomised. Instruments have been developed by prof dr P. Robert-Jan Simons and dr. Manon C.P. Ruijter MLD, as part of © Language of Learning. Version 1.2 Twynstra Gudde.

Introduction

Your development is in your own hands. You decide what to learn and how to develop yourself. When speaking about personal development, the words training and education are often heared. However, is that the best method for you? How can you make the best of your personal development?
The Language of Learning is a tool that helps to recognize, understand and identify your preferences and (usual) practice in learning. The Language of Learning gives insight in your learning and makes it possible to find a good match between the individual and the training method, in order to improve and give direction to your learning process.

Your Learning preferences

The 'Situgram' is a tool to visualize your learning preferences, in what way do you learn. Do you like to learn by visiting a lecture or by practice, or by 'plunging into a situation'? Do you prefer to learn under supervision or do you learn with or from others? Do you find it pleasant to learn with a deadline ahead or in a complex situation, or do you prefer a safe environment with time and space to experiment?
In the end, we distinguish five different ways that invite to learn, being adoption, participation, knowledge acquisition, practition and exploration.

Working method

stap 1

The situgram contains fifteen questions. All questions are followed by four of five answers. Presume, when answering the questions, an average preference (3). If an answer is (very) appealing to you, choose 4 of 5. If you consider it less pleasant or even annoying, choose 2 or 1. The more extreme your choice, the clearer your learning orientation will be after the test.

1 ——— 2 ——— 3 ——— 4 ——— 5

not al all ← average → I completely agree

1 = not at al
2 = less than average
3 = average
4 = more than average
5 = I completely agree

step 2

Repeat the scores in the white squares of the second column. This means that you sometimes have to repeat a score (twice).

Step 3

Then total the scores (step 2) per column per page.

step 4

Total the subtotals (step 3) per column per page and complete these subtotals at the top of the last page.

step 5

Then deduct 45 points from each subtotal. The scores will end up between -30 and + 30.

step 6

Fill in your personal situgram at the last page. There is an example situgram on this page that shows the scores of someone who scores 2 on participation and -5 on practition.

		step 1	step 2				
**1**	**Which circumstances help you in your development?**	0-5	1	2	3	4	5
–	When there is time and space to practice						
–	Environments with many sources of knowledge						
–	Working conditions in which I come across new, interesting questions						
–	Inspiring meetings with others						
–	Complex issues that require a quick solution						
**2**	**How do you acquire knowledge?**						
–	By looking for what works						
–	By talking to others						
–	By developing learning activities						
–	By everything I do						
**3**	**Which feelings help you in your development?**						
–	Inspiration, curiosity						
–	Safety, trust						
–	Clarity, assurance						
–	Tension, pressure of work						
**4**	**What irritates you when learning?**						
–	When something is long-winded						
–	When people withdraw from the team						
–	When there is ignorance, insufficient knowledge						
–	When I have to do something I feel unqualified for						
–	When activities lack freedom						
**5**	**What does collaboration mean for your development?**						
–	In interaction with other people I find it easier to solve problems						
–	Learning together with other people is more efficient						
–	Other people help me						
–	I consider other people a sounding board for my ideas						

step 1 0-5 | step 2 1 2 3 4 5

6 How do you deal with mistakes?

- I learn a lot from mistakes
- Mistakes keep me alert
- I try to prevent mistakes with a thorough preparation
- I do not learn a lot from mistakes

7 What do you prefer in education?

- Learning about a practical assignment
- Peer-review
- Trainings and workshops
- Company visits
- Lectures and classes

8 What or who decides the direction of your development?

- What I come across in my work or outside of my work
- The direction of development of my team
- I plan personal development with a coach, manager or trainer
- I believe that my personal development should contribute to organisational development

9 How do you organize learning when doing your job?

- I try to discuss it with others
- I read a good book once in a while
- My daily work offers enough issues to learn from
- I consciously practice new behaviour

10 What is the most important pitfall in your development?

- Taking insufficient time to think
- Taking too much time for reflection
- Finding too many things interesting
- A constant search for the truth
- Being bored too fast

step 1 step 2

0-5 1 2 3 4 5

11 Who makes you think at work?

- Experts
- Colleagues
- Critical outsiders
- Could be anyone

12 How do you respond to unfamiliar situations?

- I just plunge into it and see how it goes
- I ask others for advise
- I first do a dry run
- I try to find out as much as I can

13 What are features of the ideal supervisor?

- Didactical skills
- Skills in supervising group processes
- Practical experience
- Acuteness
- Professional knowledge

14 What kind of knowledge is important to you?

- Expertise
- Shared views
- Proven knowledge
- Knowledge giving me something to hold onto

15 What makes you think?

- Successful solutions
- Different opinions
- My own actions
- Required knowledge

Subtotal

step 3

step 4 -45 -45 -45 -45 -45

Total

Code

Ad Pa Kn Pr Ex

 © Language of Learning

Situgram

	Code	Totals
Adoption	Ad	
Participation	Pa	
Knowledge acquisition	Kn	
Practition	Pr	
Exploration	Ex	

For example: the score Ad= 1, Pa= 2, Kn=13, Pr=5, Ex=10 looks like:

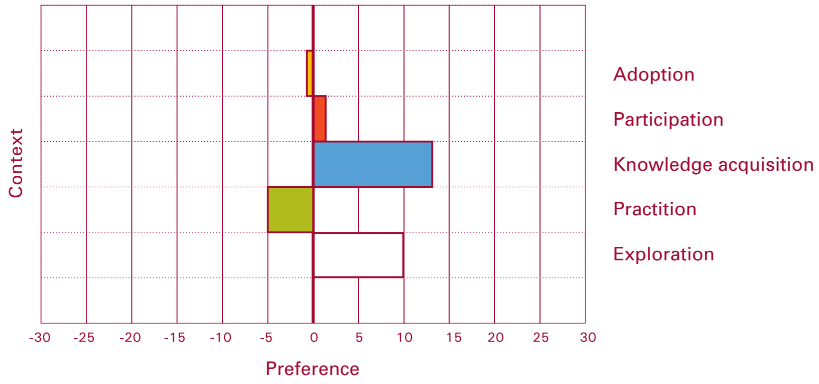

Please fill in your scores in the situgram below.

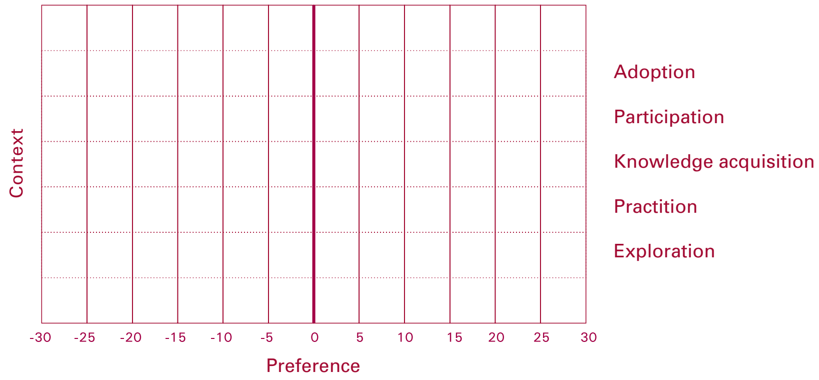

Supplement: Additional file 1: — Learning Style Questionnaire of Ruijters. [file 12913_2015_915_MOESM1_ESM.pdf]
